# From Leguminosae/Gramineae Intercropping Systems to See Benefits of Intercropping on Iron Nutrition

**DOI:** 10.3389/fpls.2019.00605

**Published:** 2019-05-14

**Authors:** Jing Dai, Wei Qiu, Nanqi Wang, Tianqi Wang, Hiromi Nakanishi, Yuanmei Zuo

**Affiliations:** ^1^College of Resources and Environmental Sciences, National Academy of Agriculture Green Development, Key Lab of Plant-Soil Interaction, MOE, China Agricultural University, Beijing, China; ^2^Graduate School of Agricultural and Life Sciences, The University of Tokyo, Tokyo, Japan

**Keywords:** iron (Fe), intercropping, rhizosphere, microorganism, exudate

## Abstract

To achieve sustainable development with a growing population while sustaining natural resources, a sustainable intensification of agriculture is necessary. Intercropping is useful for low-input/resource-limited agricultural systems. Iron (Fe) deficiency is a worldwide agricultural problem owing to the low solubility and bioavailability of Fe in alkaline and calcareous soils. Here, we summarize the effects of intercropping systems on Fe nutrition. Several cases showed that intercropping with graminaceous plants could be used to correct Fe nutrition of Leguminosae such as peanut and soybean or fruits such as *Psidium guajava* L., Citrus, grape and pear in calcareous soils. Intercropping systems have strong positive effects on the physicochemical and biochemical characteristics of soil and the microbial community due to interspecific differences and interactions in the rhizosphere. Rhizosphere interactions can increase the bioavailability of Fe with the help of phytosiderophores. Enriched microorganisms may also facilitate the Fe nutrition of crops. A peanut/maize intercropping system could help us understand the dynamics in rhizosphere and molecular mechanism. However, the role of microbiome in regulating Fe acquisition of root and the mechanisms underlying these phenomena in other intercropping system except peanut/maize need further work, which will help better utilize intercropping to increase the efficiency of Fe foraging.

## Intercropping With Graminaceous Plants Facilitates the Iron Nutrition of Leguminosae or Fruits in Calcareous Soils

Intercropping is an ancient agricultural technology that involves planting two or more crop species together. It contributes to sustainable agriculture, with higher production, higher nutrient availability (Li et al., 2014), effective weed management (Weerarathne et al., 2017), and pest control (Lopes et al., 2016) in resource-limited agricultural systems. Intercropping can improve soil quality and soil phytoavailability due to species complementarity (Li et al., 2014; Cong et al., 2015).

Iron is an essential micronutrient for plant growth. However, the solubilizing ability and bioavailability of Fe is very low in alkaline and calcareous soils, which limits plant growth and development (Guerinot and Yi, 1994). The solubility of Fe in soil is decreased with increasing pH and increasing bicarbonate concentrations, which leads to Fe deficiency in crop production on calcareous soils (Marschner, 2012). Plants have evolved two strategies to take up Fe efficiently in response to Fe deficiency: a reduction-based strategy by non-grasses (Strategy I) and a chelation-based strategy by grasses (Strategy II) (Romheld and Marschner, 1986). Under Fe-limiting conditions, Strategy I plants reinforce the reduction of Fe^3+^ to Fe^2+^, which is then transported into the root epidermis by Fe-regulated transporters (Robinson et al., 1999; Vert et al., 2002). In this process, Strategy I plants also release protons and phenolic compounds to enhance the bioavailability of Fe (Guerinot and Yi, 1994; Tsai and Schmidt, 2017). Strategy II plants secrete phytosiderophores that can bind Fe^3+^ in the rhizosphere. These complexes are then taken up by yellow stripe-like (YSL) family transporters in the roots (Curie et al., 2001; Inoue et al., 2009).

Several reports have described how intercropping systems can improve the Fe nutrition of crops (Table 1), which usually involves intercropping Strategy I plants with graminaceous Strategy II species. With the mutual benefits of intercropping systems, the Fe nutrition of the graminaceous species was also sometimes enhanced.

**TABLE 1:** The effects of intercropping systems on the iron (Fe) concentration of plant tissues.

				**Experimental**	
**Crop**	**Intercropped**	**Tissue**	**Change**	**conditions**	**References**
*Psidium guajava* L.	Maize/Sorghum	Leaf	Increased	Pot	Kamal et al.,2000
Peanut	Maize	Root, shoot, seed	Increased	Field Pot	Zuo et al., 2000, 2004; Inal et al., 2007; Dai et al.,2018
*Phaseolus vulgaris* L.	Ryegrass/oat/corn/wheat	Leaf	Not Increased^1^	Field	Omondi and Kniss,2014
*Cucumis sativus*	*Allium sativum*	Shoot	Decreased	Pot	Xiao et al.,2013
		Root	Increased		
Olive	Purple false brome/barley	Leaf	Increased	Pot	Cañasveras et al.,2014
Soybean	Maize	grain	Increased	Field	Dragicevic et al.,2015
Maize	soybean		increased		
Citrus	Barley/*Poa pratensis*/*Festuca rubra*	Root leaf	Increased	Hydroponics	Cesco et al.,2006
Grape	*Festuca ovina*	Leaf	Increased	Field	Bavaresco et al.,2010
Pear	Graminaceous species*	Leaf	#	Field	Tagliavini et al.,2000

*Mainly *Poa* spp., *Lolium* spp., and *Festuca* spp. ^1^After 1 year, the level was lower at the 8 to 16 trifoliate leaf stage, while after 2 years there was no significant difference between intercropping and monocropping. ^#^The Fe concentration of leaves was not measured, but the Fe chlorosis was amended and the SPAD value was increased in leaves.

The peanut/maize intercropping system is often used to investigate interspecies interactions between two species using different Fe uptake strategies. Peanut/maize intercropping can enhance the Fe nutrition of peanuts in calcareous soils (Zuo et al., 2000, 2004; Inal et al., 2007; Dai et al., 2018). Similarly, soybean/maize intercropping with alternating strips and organic fertilizer can increase the Fe concentration in soybean (Dragicevic et al., 2015). Although intercropping with four grass species did not increase the Fe concentration in dry bean tissues, the SPAD value was significantly higher (Omondi and Kniss, 2014). After intercropping with 12 or 24 sorghum seedlings, the Fe concentration in leaves of guava seedlings (*Psidium guajava* L.) increased by 28.2 and 52.2% in Maryut soil, respectively (Kamal et al., 2000). On three different calcareous soils, intercropping with purple false brome (*Brachypodium distachyon*) and barley (*Hordeum vulgare*) enhanced the Fe and chlorophyll concentrations in olive (*Olea europaea*) leaves (Cañasveras et al., 2014). Groundcover with grass is a promising way to enhance the Fe nutrition of fruit trees in orchards. On supplying Fe sulfate to the soil and sowing a mixture of graminaceous species along with pear (*Pyrus communis*) trees in orchards, Fe-deficiency chlorosis symptoms were alleviated (Tagliavini et al., 2000). This was also seen in citrus plants (Cesco et al., 2006) and grape (Bavaresco et al., 2010) in the presence of grass cover species. Green garlic (*Allium sativum* L.) is a non-graminaceous monocot. After intercropping with various amounts of green garlic, the root Fe concentrations of cucumber (*Cucumis sativus* L.) increased, whereas the shoot Fe concentration decreased (Xiao et al., 2013).

All of these effective cases happened in calcareous and/or alkaline soils. The low Fe bioavailability in calcareous soil is induce by pH-related effects. The bicarbonate in calcareous soil can buffer rhizosphere acidification, but also inhibits the expression of ferric reductase, iron transporters and H^+^-ATPase genes in Strategy I species such as *Arabidopsis*, pea, tomato and cucumber while has little effect on Strategy II species owing to their chelation-based strategy (Lucena et al., 2007; Marschner, 2012). Compared with graminaceous, non-graminaceous played as gainers in intercropping system on amending Fe nutrition. However, not all the graminaceous could correct the Fe nutrition of partners. The leaves of citrus and peanut did not recover from Fe deficiency chlorosis intercropped with *ys3* maize mutant plants, which is unable to release phytosiderophores (Cesco et al., 2006; Xiong et al., 2013a). It seems that a complementary strategy is necessary to increase the Fe nutrition of Strategy I plants in intercropping systems.

## Effect of Rhizosphere Change on Iron Acquisition in Leguminosae/Gramineae Intercropping Systems

The interactions in the rhizosphere affect biogeochemical cycling and nutrient availability, which affect plant growth and tolerance to biotic and abiotic stress (Philippot et al., 2013). The interactions between plant roots and the rhizosphere microbiome are critical for improving plant fitness (Zhalnina et al., 2018). Plant can secret different exudates to rhizosphere affected by environmental factors such as plant growth, nutrient availability and microorganisms (Mimmo et al., 2011), which mediate various aspects of the rhizosphere, including soil nutrient mobilization (Bakker et al., 2018; Canarini et al., 2019). The root exudates including primary metabolites (sugars, amino acids, and organic acids) can shape the rhizosphere microbiome (Badri and Vivanco, 2009). In turn, the associated microorganisms can influence plant health and growth (Huang et al., 2014). The soil microorganisms have a significant effect on nutrient availability for plants in the rhizosphere (Mimmo et al., 2014). The microorganisms can quickly utilize root exudates which affects plant nutrient foraging (Alegria Terrazas et al., 2016). Inoculation with plant growth-promoting rhizobacteria showed potential in benefiting the efficiency of nutrient acquisition of root (Pii et al., 2015).

The effects of intercropping systems on soil physicochemical and biochemical characteristics are complicated due to differences among species (Ladygina and Hedlund, 2010). In typical intercropping systems, legumes benefit soil quality by increasing soil carbon and nitrogen sequestration (Cong et al., 2015; Duchene et al., 2017). In a peanut/maize intercropping system, the available soil nitrogen and phosphorus increased in both the peanut and maize rhizosphere compared with monocropping. Furthermore, the soil urease and phosphomonoesterase activities were also improved by intercropping (Li et al., 2016b). Another field experiment showed that the pH and soil available phosphorus (Olsen-P) concentration in the rhizosphere of peanut crops decreased in the intercropping system after the vegetative stage (Guo et al., 2014). However, in seven cucumber intercropping systems, intercropping had no significant effects on physicochemical characteristics such as soil moisture, pH, and the electrical conductivity in two growing seasons (Li and Wu, 2018).

Facing Fe-limited conditions, Strategy I and II plants secrete different compounds into the rhizosphere to facilitate Fe uptake (Chen et al., 2017). The phenolic compounds, such as coumarins and flavins, secreted by Strategy I plants not only mobilize insoluble Fe but also affect the root microorganisms to promote plant health (Siso-Terraza et al., 2016; Tsai and Schmidt, 2017; Verbon et al., 2017; Stringlis et al., 2018). Different species can secrete different coumarins to better adapt to their environments (Rajniak et al., 2018). Secondary metabolites secreted from roots including antibiotics and volatiles play a key role in the performance of bacteria (Schulz-Bohm, 2018). However, the directly effect of secondary metabolites on plant nutrient foraging is barely understood. The roots of cereals such as maize secrete a class of defensive metabolites called benzoxazinoids, such as DIMBOA and DIMBOA-Glc (Hu et al., 2018a, b). They not only alter the root-associated microbiota and suppress herbivore performance but also facilitate the Fe uptake of maize. A recent study in wheat found that heterospecific combinations induced the secretion of DIMBOA (Kong et al., 2018), indicating that intercropping system may promote the secretion of DIMBOA from graminaceous contributing to Fe availability. The phytosiderophores released by graminaceous species are chelators with high affinity for ferric Fe, and can contribute to the diffusion of Fe in soil (Ma, 2005; Kobayashi and Nishizawa, 2012). The intercropped maize secreted more phytosiderophores in a peanut/maize intercropping system (Xiong et al., 2013a; Dai et al., 2018). The available Fe concentration in the rhizosphere of intercropped peanut was significantly higher than that of monocropped peanut (Guo et al., 2014).

In phosphorus-deficient soil, a maize/common bean intercropping system can efficiently increase the rhizosphere microbial biomass by regulating the C/N balance and the P availability (Latati et al., 2016; Latati et al., 2017). In peanut/maize intercropping systems, better Fe status enhances the expression of the Fe^2+^ transporter *AhDMT1* (*Arachis hypogaea* divalent metal ion transporter 1) to improve the formation of nodules and nitrogen fixation in peanut roots (Zuo et al., 2004; Shen et al., 2014). Moreover, maize root exudates can increase nodulation and stimulate nitrogen fixation by fava beans (*Vicia faba)* by promoting flavonoid synthesis in a fava bean/maize intercropping system (Li et al., 2016a). A field experiment showed that peanut/maize intercropping systems increased the abundance of nitrogen-fixing microorganisms, such as *Rhizobium hainanense*, *Rhizobium leguminosarum*, and *Frankia* (Chen et al., 2018). A pot experiment found that peanut/maize intercropping increased not only the abundance of *Rhizobium* but also *Pseudomonas* in the peanut rhizosphere (Li et al., 2018). The siderophore pyoverdine secreted by *Pseudomonas fluorescens* was beneficial to the Fe nutrition and growth of *Arabidopsis* in Fe-limited conditions (Trapet et al., 2016). The maize/legumes intercropping systems enriched the soil biodiversity and spores of AMF in the root-zone soil (Punyalue et al., 2018). Intercropping facilitated nitrogen transfer from soybeans to maize on co-inoculation with AMF, as shown by ^15^N labeling (Wang et al., 2016). AMF application in dill/common bean intercropping systems can enhance the Fe, zinc (Zn), and manganese (Mn) contents of dill and its competitive ability against weeds (Weisany et al., 2016a,b).

*Bacillus subtilis* GB03 and *Azospirillum brasilense* showed a positive effect on Fe nutrition of *Arabidopsis* and cucumber, respectively (Zhang et al., 2009; Pii et al., 2015). But about their abundance change in intercropping was rarely reported. How the plants shape their root microbiome and the effect of the specific root exudates under Fe deficiency need to be investigated. The role of microorganisms in the Fe nutrition of plant is also not fully understood. The microorganisms can increase soluble Fe in soil by decreasing soil pH through nitrification and secreting siderophores, which may be also helpful for the Fe nutrition of plant (Mimmo et al., 2014; Trapet et al., 2016). On another hand, microorganisms are competitive for Fe (Mimmo et al., 2014). In intercropping systems, more attention was paid on the effect of microorganisms on N and P turnover. Understanding the difference of effect between graminaceous and non-graminaceous on rhizosphere microbiome and their corresponding function differences can provide a new perspective for explaining the rhizosphere process in intercropping system. Knowing how the rhizosphere facilitates interactions in intercropping systems could help us to optimize plant performance by engineering the plant rhizosphere.

## Molecular Mechanism Underlying the Beneficial Effects of Intercropping on Iron Nutrition in Peanut/Maize Intercropping System

Studies of the molecular mechanisms underlying how specific intercropping interactions enhance Fe nutrition only focused on peanut/maize intercropping. In other cases, only the phenomenon was raised. The results of peanut/maize intercropping system can give us a guide to understand the Fe nutrition foraging of Leguminosae/Gramineae intercropping.

*Arachis hypogaea* iron-regulated transporter 1 (AhIRT1) was the first iron-related transporter to be isolated from the peanut. Ding et al. (2010) found higher *AhIRT1* expression during anthesis in intercropped peanut compared with monocropped peanut. In other studies, the expression of *AhIRT1* was downregulated in intercropped peanut during the reproductive stage (Xiong et al., 2013a,b; Dai et al., 2018). Further work must examine whether the intercropping interactions have a specific effect on the expression of *AhIRT1*. The expression of *AhFRO1* (*Arachis hypogaea* ferric reductase oxidase 1), which encodes a Fe(III)-chelate reductase, was affected by the growth stage in intercropping systems. During the vegetative growth of maize, *AhFRO1* was upregulated in intercropping systems while peanut was already in reproductive stage. During this period, monocropped peanut did not show Fe deficiency chlorosis. Subsequently, *AhFRO1* was downregulated in intercropping systems owing to the better Fe nutrition status. The ferric reductase activity of peanut roots showed a similar tendency, and was higher during the vegetative growth stage and lower during the reproductive growth stage of maize (Guo et al., 2014; Dai et al., 2018). The divalent metal transporter AhNRAMP1 (*Arachis hypogaea* Natural Resistance-Associated Macrophage Protein 1) is also involved in Fe uptake in peanut (Xiong et al., 2012). Both suppression subtractive hybridization and RT-PCR showed that *AhNRAMP1* was upregulated in intercropped peanut during the vegetative stage of maize. In addition, a putative MTP shared similar expression patterns with *AhNRAMP1* in an intercropping system (Dai et al., 2018).

The phytosiderophores secreted by maize chelate and solubilize Fe from the rhizosphere, and this is enhanced by specific intercropping interactions (Xiong et al., 2013a; Dai et al., 2018). The enhanced amino acid metabolism may increase the release of phytosiderophores in maize roots. Homocysteine S-methyltransferase (HMT2) and serine acetyltransferase (SAT1) are directly associated with the methionine metabolism pathway. The phytosiderophore-Fe(III) complex can be absorbed across the plasma membrane of roots by the peanut transporter protein AhYSL1 (*Arachis hypogaea* yellow stripe1-like), a homolog of maize yellow stripe 1 (Xiong et al., 2013a). Several studies have indicated that intercropping also had a positive effect on the expression of *AhYSL1*, especially during the reproductive stage (Xiong et al., 2013a; Guo et al., 2014; Dai et al., 2018). Further work must examine whether the expression of *AhYSL1* is correlated with the phytosiderophores in the rhizosphere. The soil pH and Olsen-P concentration decreased in reproductive stage under the effect of intercropping, while the available Fe concentration was the highest in rhizosphere (Guo et al., 2014). It seems that beneficial effects differ during the vegetative stage owing to dynamic changes in the rhizosphere. However, how the other factors that can influence Fe bioavailability such as rhizospheric organic compounds and the microbiome changes along with growth phase indicates that it is still an open question. Reproductive stage is a critical period of plant development which associates with sink formation (Marschner, 2012). The more nutrient demands in critical period of maize might drive the more release of phytosiderophores, which might induce the expression of *AhYSL1*.

Phytosiderophores are a class of Fe chelators that enhance adaption in the natural environment; these commonly exist in grasses, but not exclusively. 2′-Deoxymugineic acid (DMA) was detected in xylem sap from the olive (*Olea europaea* L.) (Suzuki et al., 2016). As mentioned above, intercropping with purple false brome and barley improved the Fe nutrition of olive trees. However, no study has examined whether the olive can take up DMA-Fe(III) directly, as the peanut can, in an intercropping system (Xiong et al., 2013a). Of course, homologs of the NAAT, aldo-keto reductase (similar to *DMAS*), and *YSL* family genes can be found in dicots by checking GenBank, and this is a stepping-stone for exploring the evolution of the two Fe uptake strategies. In addition, analyzing the function of phytosiderophores can help us to exploit the application of their analogs in agriculture.

The works in peanut/maize intercropping system can provide a case to understand the molecular mechanism how intercropping system benefits the non-graminaceous Fe nutrition. The further research need be taken in other intercropping systems to see if these cases can be Fe biofortification by intercropping. The driving factors of rhizosphere changes is an important task if one aims at fully understand the biological and ecological mechanism how intercropping system affect Fe turnover.

## Concluding Remarks

The previous works in intercropping system have provided different insights to uncover the effects and mechanisms of intercropping systems on Fe nutrition. Due to the interspecies facilitation of the rhizosphere, especially between Gramineae and Leguminosae, the intercropping systems show more benefits in resource-limited agricultural system including enhancing Fe nutrition. However, we still do not know how plant-microorganisms interactions regulate Fe uptake of root. The microbiome is an essential part to elucidate rhizosphere process in intercropping system. Except peanut/maize intercropping system, whether other Leguminosae/Gramineae intercropping systems can be a promising technology to facilitate Fe nutrition in calcareous/alkaline soils need be addressed further. Based on the links between molecular biology and field practices, it will contribute to a universal guide on correcting Fe deficiency chlorosis in different crops with low-input.

## Author Contributions

JD summarized and wrote the manuscript. WQ, NW, and TW all made suggestions. HN and YZ revised the manuscript. YZ provided funding for this work as corresponding author.

## Conflict of Interest Statement

The authors declare that the research was conducted in the absence of any commercial or financial relationships that could be construed as a potential conflict of interest.

## Abbreviations

AMF: arbuscular mycorrhizal fungi
bHLH: basic Helix-Loop-Helix
C/N: Carbon to nitrogen ratio
DIMBOA: 2,4-dihydroxy-7-methoxy-1,4-benzoxazin-3-one
DIMBOA-Glc: 2-(2,4-dihydroxy-7-methoxy-1,4-benzoxazin-3-one)-beta-D-glucopyranose
DMA: 2 ′ deoxymugineic acid
DMT: divalent metal ion transporter
Fe: iron
FIT: FER-like iron deficiency induced transcription factor
FRO: ferric reductase oxidase
HMT: homocysteine S-methyltransferase
IRT: iron-regulated transporter
JA: jasmonate
Mn: manganese
MTP: metal tolerance protein
NAAT: nicotianamine aminotransferase
NRAMP: natural resistance-associated macrophage protein
P: phosphorus
SAT: serine acetyltransferase
SPAD: soil-plant analysis development
YSL: the yellow stripe-Like family genes
Zn: zinc.
